# Factors that characterize oleotourists in the province of Córdoba

**DOI:** 10.1371/journal.pone.0276631

**Published:** 2022-11-03

**Authors:** José Antonio Cava Jimenez, Mª Genoveva Millán Vázquez de la Torre, Mª Genoveva Dancausa Millán

**Affiliations:** 1 Department of Agrarian Economy, Córdoba University, Córdoba, Spain; 2 Department of Quantitative Methods, Universidad Loyola Andalucía, Córdoba, Spain; 3 Department of Statistics, Córdoba University, Córdoba, Spain; Universita degli Studi di Perugia, ITALY

## Abstract

Oleotourism (olive oil tourism) is a new form of gastronomic tourism that satisfies increasingly challenging tourist demand, especially in the wake of the pandemic and for tourists who seek not only quality food products but also a safe environment to enjoy their chosen activity. Córdoba is a province in southern Spain where olives play a very important role, accounting for 50% of its cultivated area, and whose agricultural activity can be complemented with tourism due to its 189 oil mills that can welcome visitors for oil tasting. However, this type of tourism is not seeing an expected boom. This research analyzes, through a varimax analysis, the factors that attract and drive oleotourists as well as the components of such tourism. As a result, four principal components related to tourists and tourism offers were obtained, finding a high degree of satisfaction of oleotourist with the routes explored as well as a lack of knowledge of this type of tourism in international markets.

## Introduction

The COVID-19 pandemic has been a turning point in tourism; not all tourism offers are adequate, and mass tourism is not as attractive as in previous decades. Safety has become a fundamental pillar when choosing a tourist destination, as has being in nature. In Spain, since 2020 when borders were closed, tourism activity, especially foreign tourism, was paralyzed in the months of April and May, decreasing from more than 83.5 million tourists in 2019 to 18.9 million in 2020; however, in 2021, this improved, reaching 24.8 million tourists through October 2021 [1]. Faced with this decline in foreign tourism, domestic tourism has taken up the slack but not at the same levels as two years ago.

National tourists have sought rural areas, with hotels and rural houses reaching 100% occupancy during holidays such as Constitution Day, Hispanic Heritage Day, Holy Week or Christmas, while occupancy in coastal areas barely reaches 25%.

This pattern of behavior reflects the fact that vacation habits are changing and that rural areas, if they want to take advantage of this new tourist trend, must offer unique products associated with the territory that combine nature, heritage, gastronomy and tradition, that is, an enhanced tangible and intangible heritage of rural areas, among which quality agricultural products and customs or ways of cooking them are highlighted.

Spain is the leading olive oil-producing country in the world, generating 1.3 million tons in 2021 [2], and has 31 protected designations of origin (PDOs) located mainly in the south and east of the national territory, a designation that guarantees the quality of this product. By region, Andalusia has the most PDOs, with 14, followed by Catalonia with five and Castilla la Mancha with four. This food product, oil, is part of the Mediterranean diet included in UNESCO’s list of Intangible Cultural Heritage of Humanity in 2010.

Therefore, oil is not only a food product. Given its uniqueness, it has also become the basis of a new type of tourism called oleotourism, which can be defined as tourist and leisure activities and free time dedicated to the cultural discovery and enjoyment surrounding olive growing, the olive tree, oil and its territory that a person (the oleotourist) can engage in.

Oleotourism, according to Millán et al. [3], can be classified as a segment of gastronomic tourism, centered on a food product, while other authors classify it as an activity to be developed within rural tourism [4, 5] because it takes place mainly in a rural environment. Other researchers [6–8] classify it as special interest tourism (SIT) because it meets the needs of specific markets by focusing on multiple experiences and activities that are outside the scope of general interest tourism.

The aim of this study is to analyze the profile of olive oil tourists who visit PDOs in the province of Córdoba through a principal component exploratory factor analysis (EFA) and varimax rotation, as well as to determine the driving and attraction factors (push and pull) of oleotourism in this region.

## Literature review. Oil as a tourist resource

The analysis of oleotourism has three pillars: oil, gastronomy and tourism.

Olive oil is a food product that is consumed almost everywhere in the world and constitutes an essential element in Mediterranean cuisine (it is considered one of the healthiest fats in the world; it has been shown, through scientific research, that its properties help reduce cholesterol and prevent cardiovascular diseases [9]).

However, no single type of oil exists; it can vary depending on the extraction process, acidity and other parameters determined by physical-chemical analyses in a laboratory (Commission Regulation (EEC) No 2568/91 of 11 July 1991 on the characteristics of olive oil and olive-residue oil and on the relevant methods of analysis), for example, smell and taste, and can be classified into the following types:

*➢ Extra virgin olive oil*: This type of oil is extracted exclusively by mechanical procedures and has a maximum acidity of 0.8%. The lower the acidity is, the higher the quality of the fruit and the olive oil produced. This type of oil, due to its smell and taste, is mainly used in salads.
*➢ Virgin olive oil*: This type of oil is extracted in the same way as extra virgin olive oil, with the difference that the degree of acidity is usually greater than 0.8% but does not exceed 2%. That is, virgin olive oil basically differs from extra virgin olive oil in that virgin oil has defects in taste or smell. It is suitable for consumption, and it is usually used more for cooking, especially stews and fried foods.
*➢ Lampante olive oil* is the juice from the worst quality olives, usually from the last olives of the season, collected from the ground or already in the fermentation process, resulting in an oil with an acidity greater than 2% and multiple defects, thus unfit for human consumption.
*➢ Olive oil (refined + virgin)*: This type of oil contains a mixture of virgin olive oils and refined oils obtained from defective oils (lampantes) via chemical or thermal processes and has a maximum degree of acidity of 1.5%. This type of olive oil is usually tasteless and has lost a large portion of the organic compounds and natural antioxidants present in higher quality oils and is suitable for human consumption.
*➢ Pomace oil*: This type of oil contains a mixture of virgin olive oils and oils obtained by chemical processes from the solid residue of olives, called pomace, with a maximum acidity of 1.5%; this type of oil is suitable for human consumption.

The first two varieties of oils (extra virgin olive oil and virgin olive oil) are mainly used in Spanish cuisine, being highly appreciated internationally, among the 100 best chefs in the world in 2021 (Spaniards David Muñoz, ranked first, Andoni Luiz Aduriz, ranked third, and Joan Roca, ranked fourth [10]). With respect to restaurants, six Spanish restaurants are among the list of the 50 best in the world [11], two of which rank among the top 5 (Etxebarri, ranked third, and Disfrutar, ranked fifth). Therefore, Spain has good restaurants, good chefs and an excellent raw material, with the merging of these three factors being an element key to positioning Spain in the international scene as a quality gastronomic destination. In addition to Spain being part of the Mediterranean arc, where the best olive oil in the world is produced [12], and having more than 800 oil mills (where the olives are pressed to obtain the oil) that can be visited, a gastronomic and tourist product can be created that satisfies tourists’ desire for food and curiosity to learn the manufacturing process of foods.

The second pillar is gastronomy, which can be defined from two perspectives, i.e., as the set of knowledge and activities that are related to ingredients, recipes and culinary techniques and to its historical evolution and as a fondness for eating well, appreciating and enjoying good food and good restaurants. The third pillar is tourism, which comprises the travel and leisure activities carried out by a person outside their place of residence.

The intersection of these pillars, tourism, gastronomy and oil, gives rise to the concept of oleotourism. According to Henderson [13], the relationship between tourism and gastronomy can be grouped into four different lines of research: a) gastronomic tourism as a tool for the socioeconomic development of a specific destination; b) gastronomy as a tourism product; c) the relationship between gastronomy, tourism and the experience of demand; and d) the use of gastronomy as a tool for promoting and marketing a destination. The present research contributes to the line of research on the relationship between gastronomy, tourism and the experience of demand.

Apart from oleotourism, various English terms are used to link tourism to olive oil, such as agrotourism, olive oil tourism and olive-based agritourism.

Many studies focus on what motivates gastronomic tourists [14–17], as well as on gastronomy by country, e.g., France [18, 19], Italy [20], Portugal [21, 22], Croatia [23, 24] and Spain [25–27], or by products, e.g., wine [27–32], cheese [33–35], and Iberian ham [26–36]; however, there are fewer analyses of tourist offers [37–39] or demand forecasts [40–43]. In the case of oil, research on olive oil stands out. Studies that analyze the supply of olive oil include, Elias & Barbero [4], while studies that analyze the demand of olive oil include [4–65].

Tourism can be considered, in one of its many aspects, as a sociopsychological experience [66–70], although factors such as sociodemographic characteristics affect the behavior of tourists, other factors related to the subjective experience are emerging strongly to explain this complex process. In this context, motivation and satisfaction are two essential elements that determine individual behavior in the field of tourism.

A previous review of the literature on of tourists´s motivation reveals that people travel because they are “pushed” to travel for internal personal reasons or factors or because they are “attracted” by the attributes of a destination [71–74]. Push factors are more related to internal or emotional aspects, such as, rest and relaxation, adventure, social interaction, or the desire to escape. Pull factors are linked to external, situational, or cognitive aspects, such as the attributes of the chosen destination, leisure infrastructure or cultural or natural characteristics. However, these attributes of a destination can reinforce push motivations [75].

Therefore, motivation has become a meta-concept that functions as a trigger for travel behavior and determines different aspects of tourism activity with respect to (1) the reasons for traveling or “the why,” (2) the specific destination or “the where”, and (3) the outcomes or general satisfaction with the trip [76].

The relationship between motivation and satisfaction has already been studied in tourism research from different perspectives and using different methodologies (see, for example, [77–80]), and applied studies have been carried out in different market sectors ([66, 81–86], among others).

In the research on gastronomic tourism, the most expert tourists in the subject (who chose gastronomy as the main reason for visiting a destination) are more satisfied than are tourists who chose gastronomy as a secondary reason to make the trip. Their hypothesis is that organized food tours work like a well-oiled machine. Gastronomic tourists have specific needs, and tour organizers know from experience what they expect and need. Gastronomic tourists know what to expect because their main source of information is personal, not commercial. Thus, the gap between the expectations and experiences of tourists is minimized. However, this is based more on speculation than research, and new and better data are needed to fully understand this difference in satisfaction between gastronomic tourists and general tourists.

Both the satisfaction of tourists in general and their intention to repeat a trip in the future are partially determined by their rating of the different attributes of the destination [87]. In this sense, many studies explore the performance of a destination through the analysis of tourist satisfaction in different aspects of the destination [75, 88–95]. In addition, research on loyalty to a destination indicates that one of the most decisive factors in a first-time visit by tourists to an area is their satisfaction with previous stays [75, 88, 89, 96–101].

Shuo, Yeh, Ryan, Chris, Liu, and Ge Maggie [102] use a stepwise logistic regression model with repeated visit as an endogenous variable and ratings as the explanatory variables. This stepwise regression revealed very high coefficients of determination, with an adjusted R2 equal to 0.80. Conceptually, this indicates that only high satisfaction with a trip creates a strong intention to return for future visits.

## Oleotourism in the province of Córdoba

**Fig 1:**
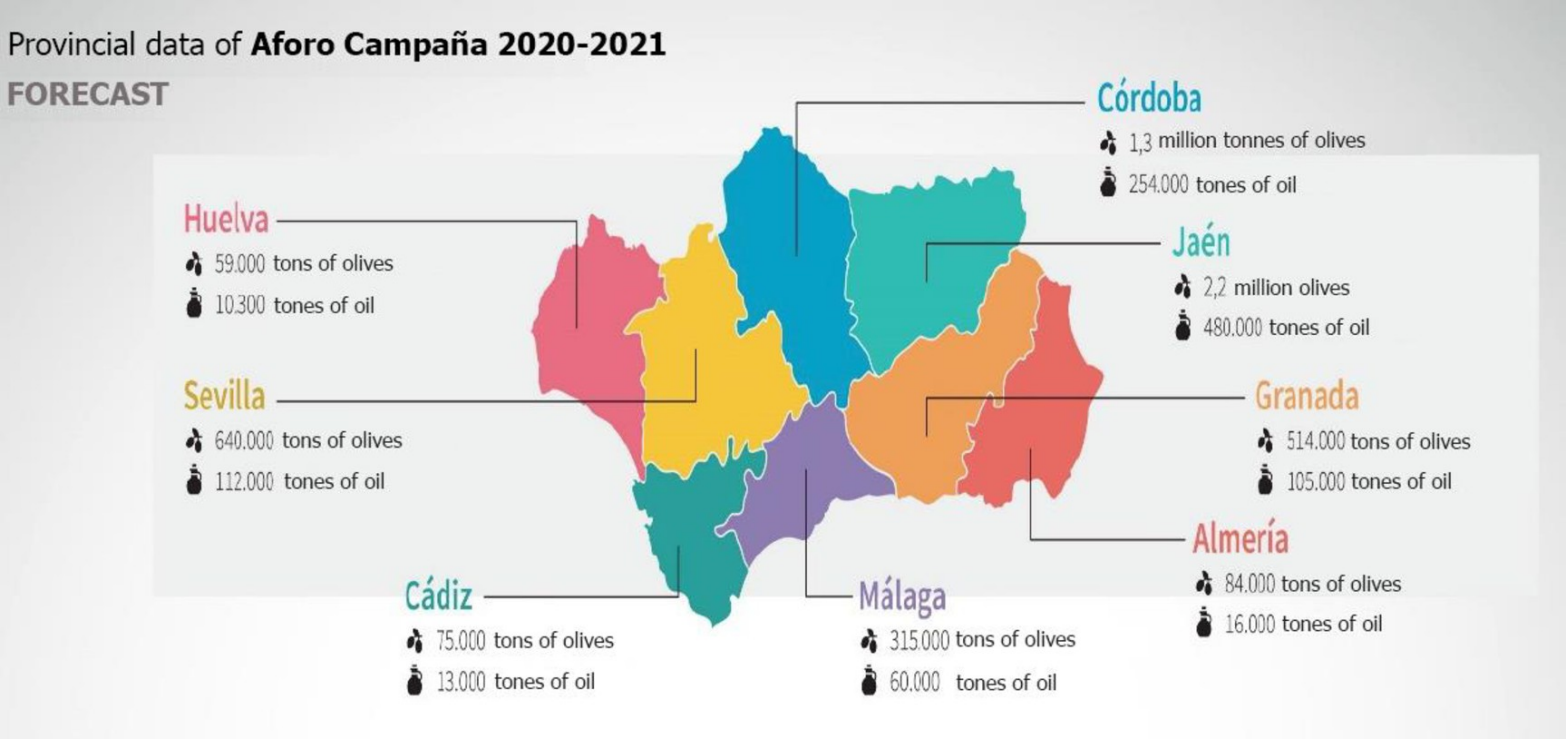
Olive cultivation area and oil production in Andalusia (2021–22 season). Source: Prepared by the authors based on information from the Agricultural and Fisheries Agency (Junta de Andalucía).

**Fig 2:**
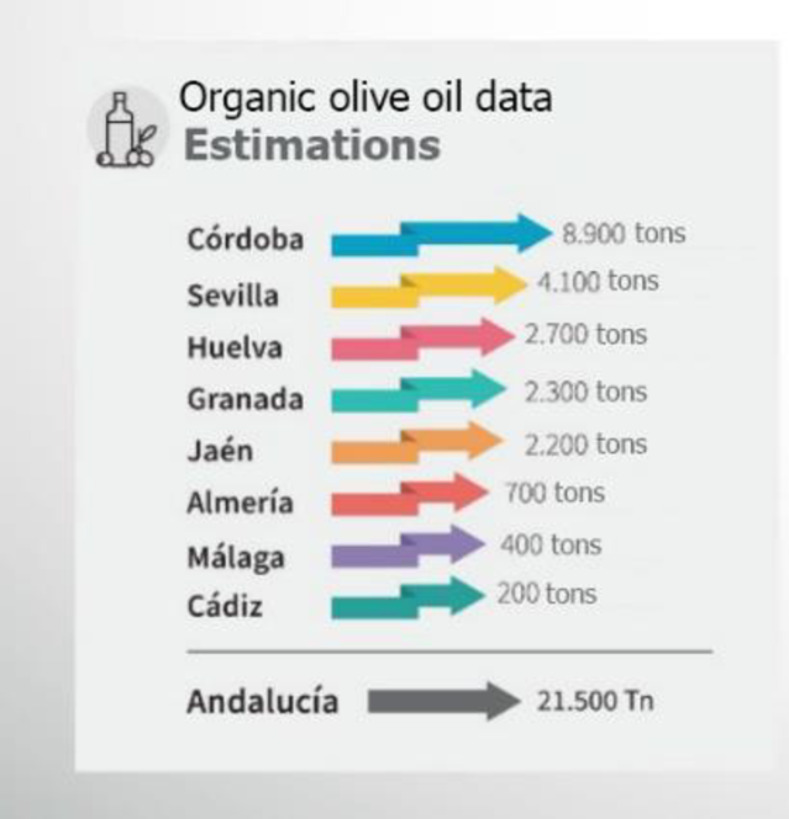
Organic oil production in Andalusia for the 2021–22 season. Source: Prepared by the authors based on information from the Agricultural and Fisheries Agency (Junta de Andalucía).

Córdoba is a province in the Autonomous Community of Andalusia and is located in southern Spain. It is the second-highest olive-producing Spanish province, with 325,589 hectares cultivated and an oil production of 254,000 tons (Fig 1), which represents 12% of the world production, and the first in terms of organic oil production (Fig 2), with 8900 tons (2021–2022 season).

**Fig 3:**
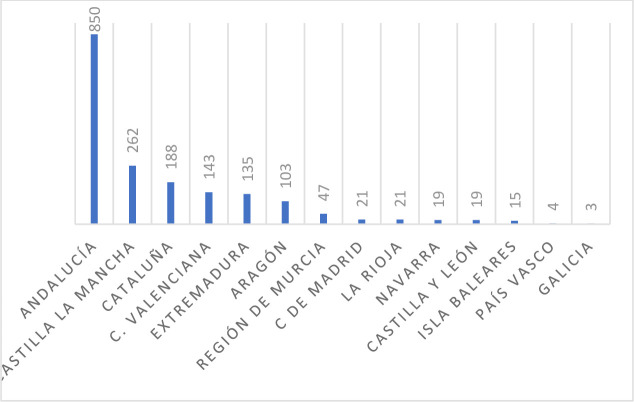
Number of oil mills by autonomous community in Spain (year 2021). Source: Prepared by the authors based on information from the Ministry of Agriculture, Fisheries and Food (MAPA).

The vast expanse of olive groves and the 189 mills (factories where olives are pressed to produce oil) of the 850 that exist in Andalusia (Fig 3) have become potential tourist attractions since Paisaje Cultural del Olivar Andaluz (Cultural Landscape of the Andalusian Olive Grove) was chosen as a UNESCO World Heritage candidate, aiming for final inclusion in the General Assembly in the summer of 2023; this designation would boost gastronomic tourism; although this sector has grown [57], it has done so more slowly than expected.

The different olive grove associations as well as the Provincial Council of Córdoba have created eight olive oil routes traversing the province of Córdoba; these routes revolve around the four designations of origin for olive oil in the province (Baena Designation of Origin, the oldest in Spain (1981), Montoro-Adamuz Designation of Origin, Priego de Córdoba Designation of Origin, and Lucena Designation of Origin) and combine the gastronomic product of oil with other destinations, such as the Torreparedones site in Baena, the Murcialagos cave, or nature tourism and sports.

However, oleotourism in Spain, despite the uniqueness and quality of the product in the Mediterranean Basin, has managed to attract only approximately 200,000 tourists, compared to the more than eight million gastronomic tourists who visit Spain mainly for gastronomy. Andalusia is visited each year by some 160,000 olive oil tourists and the province of Córdoba by some 30,000 olive oil tourists [1]. These figures indicate that in Andalusia, particulary in Córdoba, the potential of olive oil as a tourist attraction is not being sufficiently exploited.

Córdoba is a province that has four declared world heritage sites in its capital. The capital city is also one of the main destinations of cultural tourism. However, Córdoba has not yet been branded in a way that unifies the capital and province as a tourist destination, taking advantage of the synergies between the city’s cultural heritage and gastronomic products such as the oil produced in the province. As such, tourists who visit the city can be classified as excursionists, that is, they spend fewer than six hours in the city. If synergies were created between the city and the province, joint routes could be offered where oleotourism would be a tourist offering that would entice tourists to spend the night, either in the capital or in agricultural areas, and thus generate wealth since the income generated by oleotourism in the province of Córdoba does not even reach a million euros. Therefore, this work endeavors to analyze the key factors of tourism of oil in this province to improve this tourist segment.

## Materials and methods

A survey was carried out with a population composed of tourist consumers who visited any of the olive oil designations of origin in Córdoba in 2019 (oil mills, ancient olive trees, olive museums, restaurants, etc..); the objective of the survey was to learn about the profile of oleotourists. For this, a questionnaire was created that consisted of 34 questions divided into four blocks (Table 1). The first block collects personal information (age, sex, education level, marital status, etc.). The second block collects information about the route taken (how they heard about it, whether it met their expectations, what they would improve, if they came specifically for gastronomic tourism, etc.). The third block explored the motivation to participate in oleotourism (the reason for visiting the gastronomic route and using and consuming olive oil in the home). The fourth block asks oleotourists to rate their experience (regarding the services received on the route, price of the trip, hospitality and treatment received, etc.). The access by the surveyors to the olive oil route/PDO/PGI and the conduct of interviews with tourists was authorized by the managing body and owner of the DOP´s / PGI´s. Prior to the completion of the questionnaire, tourists were informed of academic purposes and anonymity in answering. Consent to take the questionnaire was verbal. At all times, the visitor’s anonymity to the olive oil route/PDO/PGI was guaranteed.

**Table 1:** Technical aspects of the survey.

	Demand survey
Population	Tourists of both sexes over 18 years of age who visited an olive oil route/PDO route in Córdoba
Sample size	214
Sampling error	±3.9%
Confidence level	95%; p = q = 0.5
Date of fieldwork	February 2019 –December 2019

With the information obtained in the survey, the following were performed.

The path model of the determinants of tourism destination image before actual visitation, which was developed by Baloglu & McCleary [65], and refers to two forces that influence a tourist destination’s image, i.e., push-pull factors resulting from two motivational forces, was used. Push forces explain the desire to travel; they are sociopsychological factors and function by impulse. Pull forces stem from external forces and depend on what tourists think about the attributes of the destination (tangible resources, e.g., oil mills, ancient olive trees, olive museums, and gastronomy, and/or intangible factors, e.g., environment and traditions). These are the attractions that motivate choices regarding destinations and/or tourist products. In this model, two basic perspectives of motivation analysis are proposed: motivation as an instinctive impulse, based on inherent needs such as the thirst for knowledge of oil manufacturing, and motivation as attraction, based on reason and emotion. In this research, a questionnaire composed of four blocks was used.

Block 1. The first block collects sociodemographic data:

- Gender
- Age
- Place of residence
- Education
- Marital status
- Employment situation
- Monthly income

Block 2. The second part of the questionnaire collects data on tourism behavior, including both qualitative and quantitative data:

- Mode of transportation
- How the destination was selected
- Organization of the trip
- Contracted services
- Daily cost of lodging services (quantitative)
- Composition of the travel group
- Duration
- Type of lodging
- Why not staying more days in Córdoba
- Visits to other cities
- Average daily expenditure per person (quantitative)
- Previous visits
- Main reason for traveling to Córdoba
- Secondary reason for traveling to Córdoba

Block 3. In the third part of the questionnaire, an importance-evaluation approach is adopted. Specifically, visitors are asked to measure the levels of importance they attribute to certain items (including both push factors and pull factors) and then their degree of satisfaction with them. Specifically, 46 items, divided into two sub-blocks, are included. The first sub-block addresses push or attraction factors:

- Historical and monumental heritage
- Local gastronomy
- Evening entertainment
- Conservation of the city environment
- Cleanliness of the city
- Ease of access—communications, roads, etc. -
- Telecommunications
- Public transportation
- Tourist information and signage
- Citizen safety
- Kindness of the people
- Quality-price ratio for lodging
- Quality-price ratio for restaurants

The second sub-block addresses pull or thrust factors:

- Visit oil mills
- Visit olive museums
- Visit ancient olive trees
- Visit cultural or historical places or events
- Enjoy nature
- Learn about a different culture

Items were included in the questionnaire based on the literature review and adapted based on elements from observations and from discussions held with different key informants to ensure a comprehensive overview of the population and the attributes of the destination.

A 10-point Likert-type scale was used to measure levels of importance and to measure levels of satisfaction; 10 represented the highest levels. In addition, following the suggestion of Ryan and Garland [103], a nonresponse option was included in the satisfaction scale because the absence of such an option can skew the results toward the midpoint of a scale.

In addition, given that satisfaction with a particular destination can be higher than a visitor’s satisfaction with the services used and the attributes of a destination [104, 105], a scale was also included to measure overall satisfaction with the visit. This measure reinforces the comprehensive (holistic) approach that is carried out in this study.

Block 4. Last, the respondents are asked to indicate if they would return to Córdoba and if they would recommend the trip to other people.

To examine the degree to which the defined indicators adequately measure the concept (construct) to be measured, an EFA was performed; this approach reduces the dimensionality of the data and is based on the analysis of the correlation between the variables. The KMO (Kaiser—Meyer—Olkin) coefficient was applied to determine that the factor analysis procedure that was performed was relevant. This statistic varies between 0 and 1. The following are commonly accepted [106, 107]:

- If KMO < 0.5, the data are not acceptable for factor analysis;
- 0.5 < KMO < 0.6 indicates a moderate degree of correlation, with moderate acceptance of the factor analysis results; and
- KMO > 0.7 indicates a high correlation, and therefore, the data are suitable for a factor analysis.

Bartlett’s sphericity test was also conducted to verify that the correlation matrix for the defined factors was not an identity matrix, which would imply a lack of correlation between the variables. If the significance level is greater than 0.05, the factorial model is not suitable for explaining the data because the null hypothesis of sphericity cannot be rejected.

The weighted correlation coefficient was applied to determine the correlation between factor loadings and the general satisfaction variable. The reliability of the instrument was determined using Cronbach’s alpha coefficient. Cronbach’s alpha is an internal consistency index that ranges between 0 and 1 and serves to verify if an instrument provides reliable measurements. The closer this statistic is to 1, the better is the reliability (≥ 0.70 indicates acceptable reliability) [108–110].

## Results

Table 2 presents the KMO test and Bartlett sphericity test results. The latter is represented by the chi-square statistic, a value (2540.392) that indicated that factor analysis was appropriate and that had a perfect significance value of zero, thus rejecting the null hypothesis. The KMO value, which measures the degree of adequacy of the sample, was 0.746, indicating that factor analysis is an adequate approach.

**Table 2:** KMO and Bartlett test results.

Kaiser–Meyer–Olkin measure of sampling adequacy		.746
Bartlett’s sphericity test	Approx. chi-square	2540.392
	Df	136
	Sig.	.000

Therefore, the questionnaire was subjected to a principal components factorial analysis, with varimax rotation, a method that minimizes the number of variables with a high load in each component, thus improving interpretability.

Component extraction: The matrix of communalities (Table 3) explains the percentage of variance in the phenomenon manifested by each variable. In this case, all the variables provide high contributions, greater than 0.5, demonstrating the high capacity of the common factors to explain the variability in each variable. Monthly income, average daily expenditure, lodging evaluation, restaurant evaluation, heritage evaluation and use of olive oil (frequency of consumption) have the highest values in the matrix; therefore, their involvement in the analysis of the resulting components will be greater.

**Table 3:** Communality matrix.

Communalities			
	Initial	Extraction	Component
Education level	1.000	.654	C2
Monthly Income	1.000	**.903**	C2
Duration of the trip	1.000	.492	C2
Average daily expenditure	1.000	**.844**	C2
Degree of satisfaction with the route taken	1.000	.720	C1
Lodging evaluation	1.000	**.*852***	C1
Restaurant evaluation	1.000	**.*809***	C1
Leisure/entertainment evaluation	1.000	.766	C3
Evaluation of the quality of the oleotourism offer	1.000	.787	C1
Evaluation of the attention and treatment received along the route	1.000	.694	C1
Evaluation of tourist signs along the route/PDO	1.000	.621	C3
Evaluation of tourist information along the route/PDO	1.000	.576	C1
Evaluation of cultural heritage of the municipality along the route	1.000	**.804**	C1
Evaluation of citizen safety along the route	1.000	.617	C3
Oil use	1.000	**.855**	C4
Oil type	1.000	.762	C4
Oil classification	1.000	.688	C4

Extraction method: principal component analysis. Column component: the component column indicates the variable to which component it belongs

**Fig 4:**
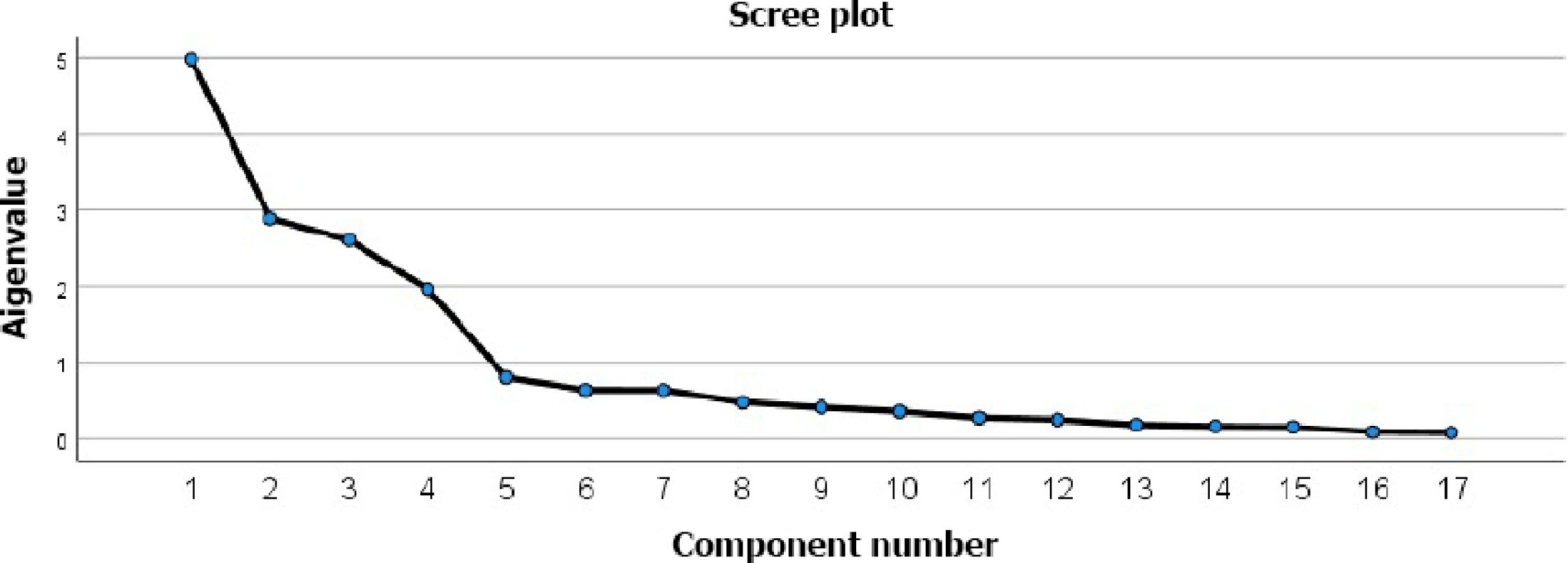
Scree plot of the factors.

Fig 4 is a scree plot showing the number of factors that provide the best explanation of the object of study. The number of factors or components appears on the X axis, which coincides with the number of items, and the eigenvalues equivalent to the variance explained by each factor are on the Y axis are. The cutoff point to establish the number of factors that are chosen as sufficient lies at inflection point 4 of the descending line that joins the various eigenvalues.

When the previous result is compared with the matrix of the total variance explained (Table 4), the first four components explain 73.209% of the total variability in the analyzed phenomenon.

**Table 4:** Matrix of total variance explained.

Total explained variance									
Component	Initial eigenvalues			Extraction sum of squared loadings			Rotation sum of squared loadings			
	Total	% variance	% accumulated	Total	% variance	% accumulated	Total	% variance	% accumulated
1	4.980	29.292	29.292	4.980	29.292	29.292	4.766	28.034	28.034
2	2.891	17.004	46.296	2.891	17.004	46.296	2.870	16.882	44.916
3	2.611	15.361	61.657	2.611	15.361	61.657	2.437	14.333	59.249
4	1.964	11.552	73.209	1.964	11.552	73.209	2.373	13.960	73.209
5	.807	4.748	77.957						
6	.642	3.775	81.732						
7	.640	3.765	85.497						
8	.480	2.821	88.318						
9	.414	2.433	90.751						
10	.360	2.117	92.868						
11	.279	1.638	94.507						
12	.253	1.490	95.997						
13	.185	1.090	97.087						
14	.163	.961	98.048						
15	.154	.905	98.952						
16	.093	.548	99.500						
17	.085	.500	100.000						

Extraction method: principal component analysis. Source: SPSS version 28.0

After factor rotation, a factorial matrix was obtained that groups the variables with respect to a certain factor and with respect to saturation. The weights or loads of the variables that compose a factor express the importance of each variable for each component in question and can even serve to identify the factor. Table 5 summarizes the factors and the highest loading variables in each one. Variables with correlation values greater than 0.6 were retained. The following factors were identified: Factor 1, “Offer”; Factor 2, “Personal”; Factor 3, “Leisure and safety”; and Factor 4, “Relationship with oil”.

**Table 5:** Rotated^a^ component matrix.

	Component			
	1	2	3	4
Opinion regarding lodging	**.909**	.086	-.136	-.014
Opinion regarding restaurants	**.898**	-.017	.050	.012
Opinion regarding the quality of the oleotourism offer	**.887**	-.013	.006	-.021
Cultural heritage of the municipality along the route	**.877**	-.085	-.150	.067
Attention and treatment received along the route	**.775**	-.048	.301	-.005
Tourist information along the route/PDO	**.742**	.035	.125	.096
Degree of satisfaction with the route taken	**.604**	-.141	.545	.196
Monthly Income	-.037	**.930**	.025	-.192
Average daily expenditure	-.065	**.916**	-.035	-.016
Education level	-.041	**.797**	-.115	.060
Duration of the trip	.086	**.646**	.029	.258
Evaluation of leisure/entertainment	.096	.007	**.870**	.022
Evaluation of tourist signs along the route/PDO	-.119	.017	**.776**	.061
Evaluation of citizen safety along the route	.096	-.078	**.773**	.064
Oil use	-.016	.021	.016	**.924**
Oil type	.018	.230	.082	**.838**
Oil classification	.117	-.127	.102	**.805**

Extraction method: principal component analysis.

Rotation method: Varimax with Kaiser normalization.^a^

a. The rotation converged in four iterations.

It is evident that the sets of variables that were grouped in the first presentation of the questionnaire have been modified to some extent. From the groups formed in the matrix, it is possible to identify each component according to the concept measured by these variables.

For *component 1* (offer evaluation), the most important variables are those concerning the offer; therefore, this is referred to as *“offer* evaluation”. Degree of satisfaction has been added, a variable that previously belonged to the personal dimension, because its coefficient is higher in this factor.

As such, the dimension offer evaluation is formed by seven evaluation items: lodging, restaurants, quality of oleotourism, heritage, attention and treatment received, route information, degree of satisfaction with the oleotourism route, and the combined synthetic perception index.

For component 2 (personal), as the matrix shows, the most essential variables are those related to the personal information of the oleotourist. The variables monthly income, average daily expenditure, education level and duration of the trip scored higher than 0.5.

In this sense, this set could still be considered personal because the variables with the highest degree of contribution are those that provide details of the economic information of oleotourists and those related to the trip, such as the average daily expenditure and duration of the trip.

The results of the matrix indicate that in the third component, “leisure and safety,” the variables related to the route are how tourists regard the leisure offer, signage and safety of the route.

**Fig 5:**
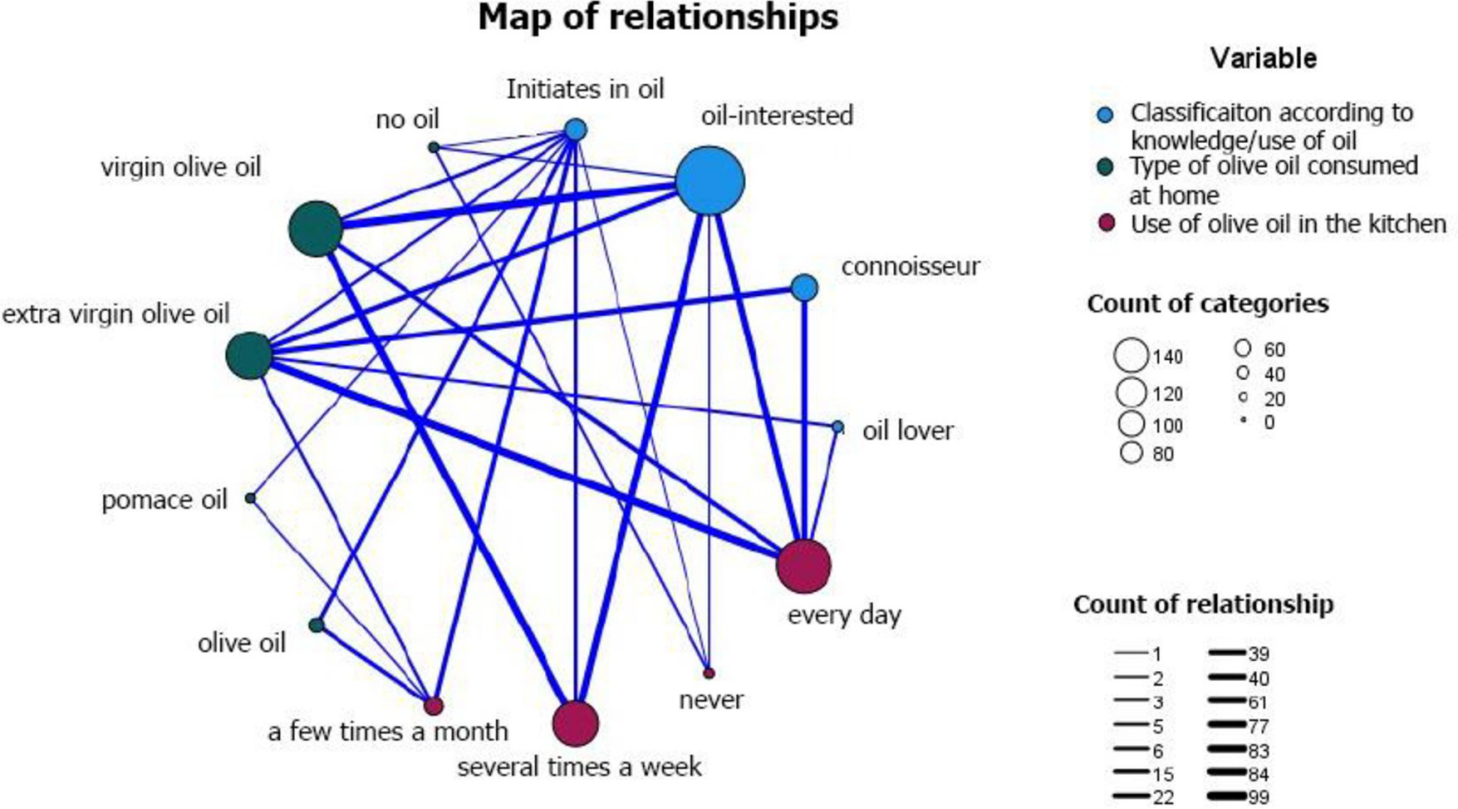
Relationship map.

The fourth dimension (oil) is formed by three items related to oleotourists’ knowledge and use of oil (Fig 5). There is a strong relationship between the type of oleotourist (oil-novice, oil-interested oil lover, and connoisseur) and the type of oil used and how often the oil is used to prepare dishes; thus those interested in oil, who are the majority of the tourists, use virgin olive oil several times a week, and connoisseurs (experts), use it daily and use a better quality extra virgin olive oil.

**Fig 6:**
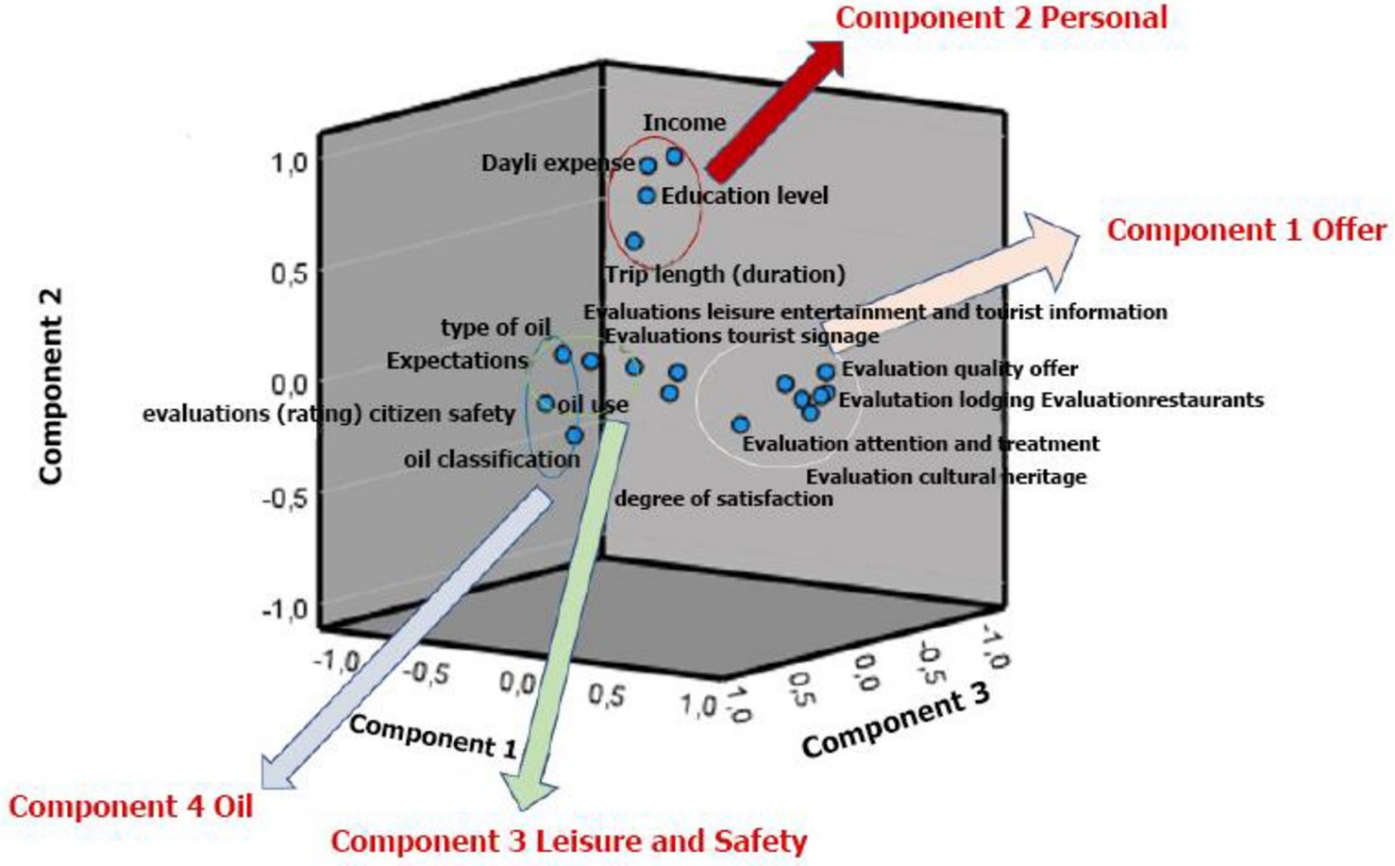
Component plot in rotated space.

Fig 6 shows a representation of each of the indicators of potentiality in the new factors created, such that each group of indicators of tourism potential is located around the axis that represents its typology of tourism resources. According to the results analyzed, some items that respond to specific dimensions do not have such an important load in the factor analysis, a finding that does not necessarily imply that these are not influencing factors; therefore, their relative importance should continue to be evaluated in a future sample.

Regarding the internal reliability index (Cronbach’s alpha), the value was 0.7784 (Table 6), with the first dimension being offer, for which there is greater homogeneity (0.913), followed by the personal dimension (0.845).

**Table 6:** Reliability statistics.

	Cronbach’s Alpha	No. elements
Total	0.778	17
Offer	0.913	7
Personal	0.845	4
Oil	0.828	3
Leisure and Safety	0.706	3

The relationship between customer satisfaction and place of origin was also analyzed; the χ2 statistic (42.775) was significant at 5%, indicating that satisfaction is related to the place of origin and that foreigners are more satisfied than are local tourists, in part because the latter know the product and are accustomed to using it, and foreign tourists are surprised by the quality of the product, especially during tasting; knowledge regarding the oil mills is something new to them because these types of venues do not exist in their country (Table 7).

**Table 7:** Pearson chi-square tests.

		Degree of satisfaction with the route taken
Place of origin	Chi-square	42.775
	Df	16
	Sig.	< .001

There is also an association between motivation to take the route and age (χ2 = 111.56, significant at 5%) (Table 8). While people from 40 to 59 years of age prefer to travel to learn about the culinary tradition of a destination, people from 18 to 30 years of age prefer to learn about the process of making oil and to visit olive oil mills.

**Table 8:** Pearson chi-square tests.

		Motivation to take the route
Age	Chi-square	111.560
	Df	8
	Sig.	.000

## Discussion

Oleotourism is a type of gastronomic tourism that is in the take-off phase in Córdoba, as in other regions of Spain [80] in part due to the lack of knowledge that gastronomic tourists, especially foreign tourists, have regarding this product (oil) [111].

The profile of oleotourists who visit the PDOs in Córdoba is very similar to that of oleotourists in Andalusia [60, 112] and that of gastronomic tourists in Mealhada-Portugal [113], i.e., between 50 and 59 years, with medium education and an upper middle income level, but in stark contrast to the demographics of the gastronomic tourists who visit Haiti, i.e.. highly educated young people with a low income [114]. The profile of oleotourists in Córdoba is also different from that of the gastronomic tourists studied by Park [115]; Robinson et al., [116]; McKercher et al., [117] and Ignatov and Smith [118], who indicate that the tourists for whom gastronomy is a relevant component in the choice of a destination are approximately 45 years old and highly educated.

The main motivation of oleotourists is to learn about the oil manufacturing process, and they usually use this product to prepare dishes. They are therefore people who are concerned about products with quality labels, who want to learn about the culture associated with olive groves, and who taste the different flavors of oils and learn about different uses; for these tourists, when they decide to take a trip, it is clear that their motivation is to learn about the local gastronomy associated with the oil, a motivation similar to that reported in a studies by Menor et al., [119] on gastronomic tourists in heritage cities. Oleotourists are concerned about sustainability and the environment; hence, they increasingly demand more organic oil and want to know about the area where it is produced.

The four components obtained in the analysis show that the supply has the greatest weight. The bivariate analysis also shows that foreign tourists are more satisfied; therefore, this work could be used by entrepreneurs to advertise oil tourism in international markets and increase the demand of oil tourists because they are the ones who most value this market niche. However, the dimensions of experience and sustainability analyzed in the work of Parrilla-González et al., [7] in the province of Jaen would have to be considered to transform Córdoba’s oleotourism into not only gastronomic tourism but also experiential tourism, given the uniqueness of the oil in the Mediterranean Basin.

## Conclusions

The gastronomy of a region, as well as the different elements that make up its dishes, is gaining strength in tourism, especially after the pandemic. Gastronomy tourists look for areas far from mass tourism destinations and with a differentiating element that guarantees product quality; this is achieved in gastronomic tourism through PDOs and geographical indications.

In this research, the profile of oleotourists in the province of Córdoba was analyzed through a factorial analysis, which revealed four factors, with one related to the personal characteristics of oleotourists, e.g., age, income level, level of education, explaining 84% of the variance. The results obtained suggest that to increase the demand for oleotourism, the communication campaigns of companies and gastronomic tourism destinations should target people with a higher education level (university studies, not secondary education as obtained in the sample) and higher incomes because they are most likely to choose Córdoba for its gastronomy. The most appropriate way of advertising would be through specialized food blogs because this medium is a more appropriate communication channel than more traditional media such as television or the press.

Another fundamental component is the offer. The results reveal how highly tourists regard restaurants and the attention and treatment received, leading to very high levels of satisfaction and Córdoba being perceived as a quality gastronomic destination associated with oil. The main problem is that Córdoba is still an unknown destination in international markets, in which it needs to be recognized.

Another important factor is the relationship between the knowledge of oleotourists regarding types of oils, the use of oils, and the type of oleotourist. Oil connoisseurs use the best quality oil, extra virgin, in their daily life and are the most satisfied tourists and who can best promote gastronomic destinations if the tourist product that is offered is a quality product.

The results of this research can help both public administrations and private entities of the province of Córdoba become the first to create, improve and promote rural development plans and promote aid for the development of tourism activity that is accessible to the small farmer who is dedicated to the olive grove and to private entities for designing a tourism product based on the olive grove and tourist demand. The objective of these efforts would be to improve the tourist offers for this very unique element and transform oleotourism into a type of special interest tourism as classified by Pulido-Fernández et al., [6].

To increase the number of olive oil tourists in Cordoba, a good marketing campaign in international markets would be needed first to publicize the gastronomic product, which is unknown by many tourists because it does not exist in their country. In addition, foreign tourists have greater purchasing power than do national tourists and are able to spend more in the area, generating more income for producers. As indicated by Pulido et al., [120], oleotourists with professional purposes show a special interest in companies such as international distributors or restaurants that can supply their products at the international scale and/or continuously across time.

The results of this study indicate that a tourist brand should be created related to the oil and all the activities that can be developed in relation to olive groves, for example, visits to ancient olive trees, olive harvesting, olive pressing, workshops for the preparation of traditional dishes, and themed meals with the reference oil, etc., in short, actions that identify the product with the territory. For this, it is first necessary that all agents, e.g., agricultural entrepreneurs, the tourism sector, the local community, and public organizations, commit to this brand because if it continues as before through individual actions, it will be difficult for Córdoba to become a quality oleotourism destination.

## Supporting information

S1 Data (SAV)

## References

[1] Instituto Nacional de Estadistica. https://www.ine.es/ (Accessed on 4 January 2022).

[2] Ministerio de Agricultura, P.y.A., Datos de las Denominaciones de Origen Protegidas (D.O.P.), Indicaciones Geográficas Protegidas (I.G.P.) y Especialidades Tradicionales Garantizadas (E.T.G.) de Productos Agroalimentarios. 2021: p. 138.

[3] Millán MG, Agudo EM. El turismo gastronómico y las Denominaciones de Origen en el sur de España: Oleoturismo. Un estudio de caso. PASOS. Revista de Turismo y Patrimonio Cultural. 2010; 8(1): 91–112.

[4] Elias SR, Barbero AC. Situación del oleoturismo y lineamientos para su desarrollo en la región del sudoeste bonaerense, Argentina. Revista interamericana de ambiente y turismo. 2017; 13(1):91–104.

[5] Ruiz I, Molina V, Martín VM. El oleoturismo como atractivo turístico en el medio rural español. Papers de turisme. 2014; 49–50: 89–103.

[6] Pulido-Fernández JI, Casado-Montilla J, Carrillo-Hidalgo I. Introducción al oleoturismo como turismo de intereses especiales. Heliyon. 2019, 5, e02975. 31872132 10.1016/j.heliyon.2019.e02975 PMC6909090

[7] Parrilla-González JA, Murgado-Armenteros EM, Torres-Ruiz F J Characterization of Olive Oil Tourism as a Type of Special Interest Tourism: An Analysis from the Tourist Experience Perspective. Sustainability. 2020; 12(15), 6008; 10.3390/su12156008

[8] Soleimani S, Bruwer J, Gross MJ, Lee R. Astro-tourism conceptualisation as special-interest tourism (SIT) field: A phenomenological approach. Curr. Issues Tour. 2019; 22: 2299–2314.

[9] Estruch R, Ros E, Martínez-González M. Primary prevention of cardiovascular disease with a Mediterranean diet. The New England journal of medicine, 2013; 369: 676–7. 23944307 10.1056/NEJMc1306659

[10] Chef, T.b., https://thebestchefawards.com/top100-the-best-chefs-2021/ (Accessed on 1 February 2022).

[11] Ltd, W.R.B.M., https://www.theworlds50best.com/.(Accessed on 25 January 2022).

[12] EVOOLEUM, La Guía EVOOLEUM World´s TOP 100 Extra Virgin Olive Oils. 2021.

[13] Henderson JC, Food tourism reviewed. British Food Journal. 2009; 111(4): 317–326.

[14] Moral-Cuadra S, et al., Relationship between consumer motivation and the gastronomic experience of olive oil tourism in Spain. Sustainability, 2020; 12(10): 4178.

[15] Cordova-Buiza F, et al., The Gastronomic Experience: Motivation and Satisfaction of the Gastronomic Tourist—The Case of Puno City (Peru). Sustainability. 2021; 13(16): 9170.

[16] González F et al., Gastronomic Motivations and Perceived Value of Foreign Tourists in the City of Oruro (Bolivia): An Analysis Based on Structural Equations. International Journal of Environmental Research and Public Health. 2020; 17(10):3618. 10.3390/ijerph17103618 PMC7277504 32455820

[17] Nicoletti S., et al., Motivations of the Culinary Tourist in the City of Trapani, Italy. Sustainability. 2019; 11(9):2686.

[18] Batat W. The role of luxury gastronomy in culinary tourism: An ethnographic study of Michelin‐Starred restaurants in France. International Journal of Tourism Research. 2021. 23(2): 150–163.

[19] Bessiere J, Tibere L. Traditional food and tourism: French tourist experience and food heritage in rural spaces. Journal of the Science of Food and Agriculture. 2013. 93(14): 3420–3425. doi: 10.1002/jsfa.6284 23794394

[20] Brandano MG, Osti L, Pulina M. How motivations and satisfaction influence wine tourists’ loyalty? An analysis of the Italian case. International Journal of Culture, Tourism and Hospitality Research, 2019. 13(1): 55–69.

[21] Andrade-Suárez M, Caamaño-Franco I. The Relationship between industrial heritage, wine tourism, and sustainability: A case of local community perspective. Sustainability. 2020. 12(18): 7453.

[22] Fernandes C, Richards G. Developing gastronomic practices in the Minho region of Portugal. Acta geographica Slovenica. 2021; 61(1): 141–152.

[23] Ivanović S, Klimoska AM, Milojica V. Measuring satisfaction and experienced sentiments of website users when exploring croatian gastronomic tourist offer. Tourism in South East Europe. 2019. 5: 335–348.

[24] Drpić D,. Klimoska AM, Manestar D. Contribution of gastronomic tourism for achieving competitiveness of croatian coastal destinations. Faculty of Tourism and Hospitality Management in Opatija. Biennial International Congress. Tourism & Hospitality Industry. 2020: 107–120.

[25] Dancausa MG. Millán MG, Hernández R. Analysis of the demand for gastronomic tourism in Andalusia (Spain). PloS one.- 2021. 16(2): e0246377. doi: 10.1371/journal.pone.0246377 33544734 PMC7864416

[26] Cava JA. Millán MG. Hernández R. Analysis of the Tourism Demand for Iberian Ham Routes in Andalusia (Southern Spain): Tourist Profile. Sustainability. 2019. 11(16): 4278.

[27] Carral EV, Del Río M, López Z. Gastronomy and Tourism: Socioeconomic and Territorial Implications in Santiago de Compostela-Galiza (NW Spain). International Journal of Environmental Research and Public Health, 2020. 17(17): 6173. 10.3390/ijerph17176173 PMC7504474 32854422

[28] Crespi-Vallbona M, Mascarilla-Miró O. Wine lovers: Their interests in tourist experiences. International Journal of Culture, Tourism and Hospitality Research, 2020;14(2) 239–258. doi: 10.1108/IJCTHR-05-2019-0095:.

[29] Madeira A, Correia A, Filipe A. Wine Tourism: Constructs of the Experience. In Trends in Tourist Behavior- New Products and Experiences from Europe. 2019: 93–108.

[30] Wang C. The Worlds approach” to gastronomic tourism: The case of wine tourism in Japan, in The Routledge Handbook of Gastronomic Tourism. 2019, Routledge. p. 385–392.

[31] Duarte A, Martens W, Ong JLT, Food tourism development in wine regions: perspectives from the supply side. Current Issues in Tourism. 2021: 1–19. 10.1080/13683500.2021.1935791

[32] Serra-Cantallops A, Ramón-Cardona J, Vachiano M. Increasing Sustainability through Wine Tourism in Mass Tourism Destinations. The Case of the Balearic Islands. Sustainability, 2021. 13(5): 2481.

[33] Folgado-Fernández JA, Di-Clemente E, Hernández-Mogollón JM, Food festivals and the development of sustainable destinations. The case of the cheese fair in Trujillo (Spain). Sustainability. 2019; 11(10): 2922.

[34] Fusté-Forné F. Developing cheese tourism: a local-based perspective from Valle de Roncal (Navarra, Spain). Journal of Ethnic Foods. 2020; 7(1):1–9.

[35] Ermolaev V A, Yashalova NN, Ruban D A Cheese as a tourism resource in Russia: the first report and relevance to sustainability. Sustainability. 2019; 11(19): 5520.

[36] Pizarro A, Šadeikaitė G, García FJ.The World of Iberian Ham and its Tourist Potential in the Sierra De Huelva (Andalusia, Spain). 2020.

[37] Tafel MC, Szolnoki G. Relevance and challenges of wine tourism in Germany: a winery operators’ perspective. International Journal of Wine Business Research. 2020; 33(1): 60–79

[38] Virgil N, Simona S. Gastronomic tourism, an opportunity for diversifying the tourist offer in the Sibiu area. Вісник Киiвського нацiонального унiверситету iм. Тараса Шевченка. Серiя: Економiка. 2019;1 (202):48–54.

[39] Hernandez RD, Dancausa MG. Turismo gastronómico. La gastronomía tradicional de Córdoba (España). Estudios y perspectivas en turismo. 2018. 27(2): 413–430.

[40] Di Clemente E. Las variables experienciales como determinantes de la calidad de vida, la satisfacción y la lealtad del turista en el contexto del turismo gastronómico. 2017. Universidad de Extremadura.

[41] Henche BG. Los mercados de abastos y su comercialización como producto de turismo de experiencias. El caso de Madrid. Cuadernos de turismo. 2017; (39): 167–189.

[42] Gardel C. Análisis de la demanda turística desde el punto de vista de la motivación y satisfacción de su gastronomía El caso Salinas (Ecuador). HOLOPRAXIS. 2018. 2(1): 225–249.

[43] Carvache-Franco M., et al., The Tourist Demand from the Perspective of the Satisfaction, Attitude and Preferences of Their Gastronomy: The Case of Salitre (Ecuador. Turismo y Sociedad. 2018; XXII:151–156. 10.18601/01207555.n22.08.

[44] Aybar R. Proyecto Oleoturismo: una red europea para la promoción de la cultura del olivo. 2004.

[45] Millán MG, Agudo EM, Agudo I. Oil-tourism in the South-east of Spain: The necessity of coordinating the tourist activity with the agrarian one for the development of the rural areas. Panorama Socioeconómico.2010. 28(41): 116–124.

[46] Millan MG, Morales EJ, Pérez LM Olive oil tourism as a vehicle for rural development in the province of Cordoba. Turismo & Desenvolvimento.2010.; 2(13): 739–748

[47] Millan MG, Morales EJ, Agudo EM El oleoturismo como motor de desarrollo rural: La denominación de origen de Montoro-Adamuz. Mundo Agrario.2010; 11(21): 1–27

[48] Millán MG, Pérez L, Morales EJ. La Ruta del aceite de oliva en la DOP Baena como opción estratégica de desarrollo. Revista de Economía, Sociedad Turismo y Medioambiente—RESTMA.2010; 10: 33–52

[49] Quesada JM, Molina V, Ruiz I. Oleoturismo en España: Potencialidad de éxito internacional en escenarios actuales. Global Conference on Business and Finance Proceedings. 2010; 5(2): 1534–1560

[50] Alonso AD, Northcote J. The development of olive tourism in Western Australia: A casestudy of an emerging tourism industry. International Journal of Tourism Research.2010; 12(6): 696–708

[51] Millan MG, Agudo EM, Morales EJ, Análisis de la oferta y la demanda de oleoturismo en el sur de España: Un estudio de caso. Cuadernos de Desarrollo Rural. 2011; 8(67): 181–202

[52] López-Guzmán T, González-Fernández V. Socioeconomic development in rural areas through the creation of tourist routes: An olive tourism approach in the Jaen province (Spain). European Journal of Tourism, Hospitality and Recreation. 2011 2(2): 5–18

[53] Millan MG, Morales EJ. Denominaciones de Origen Protegidas (DOP) y turismo gastronómico: una relación simbiótica en Andalucía. Gran Tour. 2012; 6: 101–121

[54] Murgado E. M. Turning food into a gastronomic experience: olive oil tourism. Options Mediterranéennes. 2013; 106: 97–109. http://om.ciheam.org/article.php?IDPDF=6809

[55] Campón-Cerro AM, Di-Clemente E, Hernández-Mogollón J. M., De Salvo P, Calzati V. Olive oil tourism in Southern Europe: Proposals for tourism development of olive grove rural areas. Turismo & Desenvolvimento. 2014. 21(22): 63–73

[56] Hernández JM, Di Clemente E, López-Guzmán T, Campón AM. Oleoturismo: Una nueva oportunidad de desarrollo turístico y territorial para Extremadura. En: Martín D, López JM Aceite de oliva virgen, saber y sabor de Extremadura. Consejería de Medio Ambiente y Rural de la Junta de Extremadura, Badajoz, 327–335

[57] Millán GL, Amador L, Arjona JM. El oleoturismo: una alternativa para preservar los paisajes del olivar y promover el desarrollo rural y regional de Andalucía (España). Revista de Geografía Norte Grande. 2015; (60): 195–214.

[58] Ruiz I. Análisis cuantitativo y cualitativo del significado del aceite de oliva. Una aproximación desde el punto de vista del consumidor. Servicios de Publicación de la Universidad de Granada, Granada. 2010.

[59] Molina V, Quesada J M, Ruiz I. Potencial del oleoturismo como diversificación económica del sector cooperativo agrario: el caso español. Revista de Ciencias Sociales. 2011; 17(3): 533–541

[60] Cañero PM, López-Guzmán TJ, Moral S, Orgaz F. Análisis de la demanda del oleoturismo en Andalucía. Revista de Estudios Regionales. 2015; (104): 133–149

[61] Orgaz F, Moral S, López-Guzmán T, Cañero P. Estudio de la demanda existente en torno al oleoturismo. El caso de Andalucía. Cuadernos de Turismo. 2017; (39): 437–453

[62] Millán-Vázquez G, Pablo-Romero M, Sánchez J. Oleotourism as a Sustainable Product: An Analysis of Its Demand in the South of Spain (Andalusia). Sustainability. 2018; 10(1), 1–19

[63] Murgado-Armenteros E M, Parrilla-González J A, Medina-Viruel M J. What does the olive oil tourist value at the destination? A criterion for olive oil tourism segmentation. International Journal of Gastronomy and Food Science. 2021; 25, 100378.

[64] Hernández-Mogollón J M, Di-Clemente E, Folgado-Fernández J A, Campón-Cerro AM. Olive oil tourism: state of the art. Tourism and hospitality management. 2019. 25(1), 179–207.

[65] Baloglu S, McCleary K. U.S. International Pleasure Travelers’ Images of Four Mediterranean Destinations: A Comparison of Visitors and Nonvisitors. Journal of Travel Research—J TRAVEL RES. 1999; 38: 144–152.

[66] Del Bosque IAR, San Martín H, Collado J. The role of expectations in the consumer satisfaction formation process: Empirical evidence in the travel agency sector. Tourism management. 2006. 27(3): 410–419.

[67] Castaño JM. Psicología social de los viajes y del turismo. 2005: Paraninfo.

[68] Ross ELD, Iso-Ahola SE. Sightseeing tourists’ motivation and satisfaction. Annals of tourism research. 1991. 18(2): 226–237.

[69] Wacker W. Changing demands. Journal of Advertising Research. 1996; 36(1): 31–34.

[70] Gil Á. Sociología del Turismo. 2003.

[71] Crompton JL. Motivations for pleasure vacation. Annals of tourism research. 1979. 6(4): 408–424.

[72] Dann GM. Anomie, ego-enhancement and tourism. Annals of tourism research, 1977; 4(4): 184–194.

[73] Dann GM. Tourist motivation an appraisal. Annals of tourism research. 1981. 8(2): 187–219.

[74] Uysal M, Jurowski C. Testing the push and pull factors. Annals of tourism research. 1994; 21(4): 844–846.

[75] Yoon Y, Uysal M. An examination of the effects of motivation and satisfaction on destination loyalty: a structural model. Tourism management, 2005. 26(1): 45–56.

[76] Moreno A., et al., Aproximación psicosocial a la motivación turística. Estudios turísticos. 2003; 158: p. 5–42.

[77] Ibrahim EE, Gill J. A positioning strategy for a tourist destination, based on analysis of customers’ perceptions and satisfactions. Marketing intelligence & planning, 2005; 6(2):131–151. 10.1108/JTA-05-2019-0019

[78] García ML, Picos AP. La calidad percibida como determinante de tipologías de clientes y su relación con la satisfacción: Aplicación a los servicios hoteleros. Revista europea de dirección y economía de la empresa. 2009; 18(3): 189–210.

[79] Oliver RL. A cognitive model of the antecedents and consequences of satisfaction decisions. Journal of marketing research. 1980; 17(4): p. 460–469.

[80] Severt D, et al., Examining the motivation, perceived performance, and behavioral intentions of convention attendees: Evidence from a regional conference. Tourism management. 2007; 28(2): p. 399–408.

[81] Fernández MD, Picos AP. Predicciones en el nivel de satisfacción percibida por los turistas a partir de variables motivacionales y de valoración de la visita. ICE, Revista de Economía. 2005; (821):241–256.

[82] Fernández MD, Picos AP. Determinantes de la satisfacción percibida en el turismo rural. in Turismo en espacios rurales: 8.° Congreso de turismo universidad y empresa. 2006. Tirant lo Blanch.

[83] Sanchis MG, Saura IG. Expectativas, satisfacción y lealtad en los servicios hoteleros. Un enfoque desde la cultura nacional. Papers de Turisme. 2012; (37–38):7–25.

[84] Lee CK, Lee YK, Wicks BE. Segmentation of festival motivation by nationality and satisfaction. Tourism management. 2004; 25(1): p. 61–70.

[85] Brenner EL. La motivación turística: el caso de la región de las aguas termales de Goiás, Brasil. Boletín de La Asociación de Geógrafos Españoles. 2006; 42: 303–316

[86] Qu H, Ping EWY, A service performance model of Hong Kong cruise travelers’ motivation factors and satisfaction. Tourism management. 1999; 20(2): 237–244.

[87] Alegre J, Garau J. Tourist satisfaction and dissatisfaction. Annals of tourism research. 2010; 37(1): 52–73.

[88] Alegre J, Cladera M. Repeat visitation in mature sun and sand holiday destinations. Journal of travel research. 2006; 44(3): 288–297.

[89] Baker DA Crompton JL. Quality, satisfaction and behavioral intentions. Annals of tourism research. 2000. 27(3):785–804.

[90] Crompton JL Love LL. The predictive validity of alternative approaches to evaluating quality of a festival. Journal of travel research. 1995. 34(1): 11–24.

[91] Danaher PJ, Arweiler N. Customer satisfaction in the tourist industry: A case study of visitors to New Zealand. Journal of travel research. 1996; 35(1): 89–93.

[92] Kozak M, Rimmington M., Measuring tourist destination competitiveness: conceptual considerations and empirical findings. International Journal of Hospitality Management. 1999; 18(3): 273–283.

[93] Kozak M. Destination benchmarking. Annals of tourism research. 2002; 29(2): 497–519.

[94] Murphy P, Pritchard MP, Smith B. The destination product and its impact on traveller perceptions. Tourism management. 2000; 21(1): 43–52.

[95] Pizam A, Ellis T. Customer satisfaction and its measurement in hospitality enterprises. International journal of contemporary hospitality management. 1999; 11(7): 326–339.

[96] Appiah-Adu K, Fyall A, Singh S. Marketing culture and customer retention in the tourism industry. The Service Industries Journal. 2000; 20(2):95–113.

[97] Bigne JE, Sanchez MI, Sánchez J. Tourism image, evaluation variables and after purchase behaviour: inter-relationship. Tourism management. 2001; 22(6):607–616.

[98] Caneen JM. Cultural determinants of tourist intention to return. Tourism Analysis. 2003, 8(2): 237–242.

[99] Marques C, da Silva RV, Antova S. Image, satisfaction, destination and product post-visit behaviours: How do they relate in emerging destinations?. Tourism Management. 2021; 85, 104293.

[100] Kozak M. Repeaters’ behavior at two distinct destinations. Annals of tourism research. 2001; 28(3): 784–807.

[101] Kozak M. Measuring tourist satisfaction with multiple destination attributes. Tourism analysis. 2003; 7(3–4): 229–240.

[102] Shuo YSS, Ryan C, Liu GM: Taoism, temples and tourists: The case of Mazu pilgrimage tourism. Tourism management. 2009; 30(4): 581–588.

[103] Ryan C, Garland R. The use of a specific non-response option on Likert-type scales. Tourism management. 1999; 20(1): 107–113.

[104] Truong TH, Foster D. Using HOLSAT to evaluate tourist satisfaction at destinations: The case of Australian holidaymakers in Vietnam. Tourism Management—TOURISM MANAGE. 2006; 27: 842–855.

[105] Yu L, Goulden M. A comparative analysis of international tourists’ satisfaction in Mongolia. Tourism Management. 2006; 27: 1331–1342.

[106] Fernández-Altuna MA, López-Ortega M. López-López E, Gutiérrez-Rayón D, del Prado A M, Meléndez CA. Validación de un cuestionario para la determinación de factores de riesgo físico, alimentarios y de descanso para enfermedades crónico-degenerativas en población adulta de la Ciudad de México. Salud en Tabasco. 2017; 23(1–2): 34–43.

[107] Ronquillo L, Aranda C, Pando M. Validación de un instrumento de evaluación del desempeño en el trabajo. Revista Iberoamericana de Psicología, 2013; 6(1):25–32.

[108] Santoro E, Hernandez R, Fernández-Collado C, Baptista L. Research Methodology (4th Edic). McGraw Hill: DF, Mexico, Mexico, 2006.

[109] Gonzalez J, Pazmiño M. Calculation and interpretation of Cronbach’s Alpha for the case of validation of the internal consistency of a questionnaire, with two possible Likert scales. Journal publishing. 2015; 2(1): 62–67.

[110] Rodriguez-Rodriguez J, Reguant-Álvarez M. Calculate the reliability of a questionnaire or scale using the SPSS: Cronbach’s alpha coefficient. REIRE Revista d’Innovació i Recerca en Educació. 2020; 13(2): 1–13.

[111] Hernández-Mogollón J.M., et al., Olive oil tourism: state of the art. Tourism and hospitality management. 2019; 25(1): 179–207.

[112] Pulido-Fernández JI, Casado-Montilla J, Carrillo-Hidalgo I. Understanding the Behaviour of Olive Oil Tourists: A Cluster Analysis in Southern Spain. Sustainability. 2020; 12(17): 6863.

[113] Oliveira S. La gastronomía como atractivo turístico primario de un destino: El Turismo Gastronómico en Mealhada—Portugal. Estudios y perspectivas en turismo. 2011; 20: 738–752.

[114] Orgaz-Agüera F. López-Guzmán T. Análisis del perfil, motivaciones, y valoraciones de los turistas gastronómicos. El caso de la República Dominicana. Ara: Revista de Investigación en Turismo. 2017; 5(1): 43–52.

[115] Park K. Ethnic Foodscapes: Foreign Cuisines in the United States. Food Culture and Society An International Journal of Multidisciplinary Research. 2017; 20(3): 365–393.

[116] Robinson R, Getz D, olnicar S. Food tourism subsegments: A data-driven analysis. International Journal of Tourism Research. 2018; 20(3):367–377.

[117] McKercher B, Okumus F, Okumus B. Food Tourism as a Viable Market Segment: It’s All How You Cook the Numbers! Journal of Travel & Tourism Marketing. 2008; 25:137–148.

[118] Ignatov E, Smith S. Segmenting Canadian Culinary Tourists. Current Issues in Tourism. 2006; 9: 235–255.

[119] Menor-Campos A, et al., Gastronomía local, cultura y turismo en Ciudades Patrimonio de la Humanidad: el comportamiento del turista extranjero. Investigaciones Turísticas. 2022; 23: 140–161.

[120] Pulido-Fernández JI, Casado-Montilla J, Carrillo-Hidalgo I, Análisis del comportamiento de la demanda de oleoturismo desde la perspectiva d ela oferta. Revista de Investigaciones Turísticas. 2021; 21:67–85. I.

